# Evaluation of haematological parameters in haemolytic anaemia caused by tick‐borne pathogens in grazing cattle

**DOI:** 10.1002/vms3.1434

**Published:** 2024-04-03

**Authors:** Youngjun Kim, Ji‐Young Ku, Youngwoo Jung, Young‐Hwan Lim, Min‐Jeong Ji, Yu‐Jin Park, Hyung‐Chul Cho, Kyoung‐Seong Choi, Jinho Park

**Affiliations:** ^1^ Department of Veterinary Internal Medicine College of Veterinary Medicine Jeonbuk University Iksan Republic of Korea; ^2^ Department of Animal Hospital, Hanwoo (Korean Indigenous Cattle) Genetic Improvement Center National Agricultural Cooperative Federation Seosan Republic of Korea; ^3^ Department of Animal Science and Biotechnology, College of Ecology and Environmental Science Kyungpook National University Sangju Republic of Korea

**Keywords:** grazing cattle, haemolytic severe anaemia, indirect bilirubin, L‐lactate, tick‐borne pathogens

## Abstract

**Background:**

No tick‐borne pathogens (TBPs) causing haemolytic anaemia in cattle have been reported, except *Theileria orientalis* and complete blood count (CBC) profile is the only haematological parameter to determine the severity of regenerative haemolytic anaemia.

**Objectives:**

To identify the causative agents of TBP‐induced haemolytic anaemia and determine haematological parameters that indicate haemolytic anaemia in grazing cattle.

**Methods:**

Eighty‐two Korean indigenous cattle (Hanwoo) were divided into two groups: grazing (*n* = 67) and indoor (*n* = 15) groups. CBC and serum biochemistry were performed. PCR was conducted using whole blood‐extracted DNA to investigate the prevalence of TBPs.

**Results:**

TBP‐induced haemolytic anaemia was observed in the grazing group. In grazing cattle, co‐infection (43.3%, 29/67) was most frequently detected, followed by *T. orientalis* (37.6%, 25/67) and *Anaplasma phagocytophilum* infections (1.5%, 1/67). In indoor cattle, only co‐infection (20%, 3/15) was identified. Grazing cattle exhibited regenerative haemolytic anaemia with marked monocytosis, mild neutropenia, and thrombocytopenia. According to grazing frequency, the 1st‐time grazing group had more severe anaemia than the 2nd‐time grazing group. Elevations in indirect bilirubin and L‐lactate due to haemolytic anaemia were identified, and correlations with the respective markers were determined in co‐infected grazing cattle.

**Conclusions:**

Quantitative evaluation of haematocrit, mean corpuscular volume, and reticulocytes (markers of regenerative haemolytic anaemia in cattle) was performed for the first time. Our results show that, in addition to *T. orientalis*, *A. phagocytophilum* is strongly associated with anaemia. The correlation between haemolytic anaemia severity and haematological parameters (indirect bilirubin, reticulocytes, and L‐lactate) was confirmed.

## Graphical abstract

In grazing Korean indigenous cattle, haemolytic anaemia caused by *Theileria orientalis* and *Anaplasma phagocytophilum* has been observed.

Reticulocytosis, a marker of regeneration, was quantitatively expressed with an increase in indirect bilirubin and a decrease in haptoglobin.

In addition, indirect bilirubin, L‐lactate and reticulocytes were correlated with the severity of anaemia after HCT.

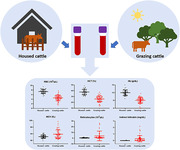

## 1 INTRODUCTION

Hard ticks are vectors of various pathogens, such as bacteria, viruses and protozoa, and cause serious diseases, such as anaplasmosis and theileriosis in cattle (Agina et al., 2020; Aubry & Geale, 2011; El‐Alfy et al., 2022). The *Anaplasma* genus comprises six species based on host cell tropism. Three species parasitise red blood cells (erythrocytic *Anaplasma*): *A. marginale*, *A. centrale* and *A. ovis*. The other three species infect neutrophils, monocytes, and platelets: *A. phagocytophilum*, *A. bovis* and *A. platys*, respectively (Dumler & Walker, 2001). Bovine anaplasmosis is caused by *A. marginale*, *A. centrale*, *A. phagocytophilum*, and *A. bovis*, causing extravascular haemolytic anaemia (Kocan et al., 2000; Kuttler, 1984). *Theileria* parasites are divided into two species based on their ability to transform host cells. *Theileria annulata* and *T. parva* are distributed in East and Central Africa and considered the most pathogenic species in cattle. However, other *Theileria* species, such as *T. mutans*, *T. velifera* and *T. orientalis*, that do not transform host cells, usually cause a less severe disease in cattle (Sivakumar et al., 2014). Bovine theileriosis in the Republic of Korea (ROK) is mainly associated with *T. orientalis* infection, and its prevalence is increasing (Alkathiri et al., 2023; Park et al., 2017).

Anaemia in cattle can be regenerative or non‐regenerative and regenerative anaemia is further divided into haemorrhagic and haemolytic anaemia (Jones & Allison, 2007; Schalm et al., 2010). Bovine regenerative anaemia can be diagnosed by increased polychromatinisation of red blood cells (RBCs) on a blood smear or by identification of basophilic stippling. The most accurate way to diagnose regenerative anaemia in cattle is through reticulocytosis. Haemolytic anaemia in cattle may be caused by protozoan infections (*Theileria*/Babesia spp.), bacterial infections (*Anaplasma* spp., *Mycoplasma* spp., *Clostridium* spp., and *Leptospira* spp.), toxicosis (copper, onion, *Brassica* spp., and red maple leaves) or immune‐mediated haemolytic anaemia (Chase et al., 2017; Jones & Allison, 2007; Schalm et al., 2010).

Oxygen delivery is a product of cardiac output and arteriovenous oxygen content. When anaemia occurs, haemoglobin (Hb) decreases, causing hypoxia in peripheral tissues and a compensatory increase in cardiac output (Anand & Florea, 2001; Metivier et al., 2000). Anaemia should be treated carefully because hypoxia occurs in peripheral tissues and the heart is overloaded. Anaemia ‐induced hypoxia of peripheral tissues can lead to an increase in organic acids, such as lactate, resulting in metabolic acidosis (Constable et al., 2016; Lee et al., 2015). The severity of metabolic acidosis; an increase in the anion gap; and most importantly, an increase in lactate may be used to assess the severity of anaemia (Constable et al., 2016; Hardy, 2004; Magdesian et al., 2006).

In haemolytic anaemia, clinicopathological changes accompany RBC destruction. During intravascular haemolysis, RBCs within blood vessels are destroyed, and Hb and lactate dehydrogenase (LDH) are released into the blood. Haptoglobin (Hp) binds to Hb, and unbound Hb is transported to the kidneys where it is broken down, causing indirect bilirubinemia (Russell & Roussel, 2007; Smith et al., 2020; Stockham & Scott, 2008a). Destruction of RBCs by macrophages in the spleen, an increase in indirect bilirubin (a metabolite of Hb) and splenomegaly have been reported in extravasation haemolysis (Smith et al., 2020; Stockham & Scott, 2008a; Zachary & McGavin, 2017).

Only few studies showing the presence or increase in reticulocytes as evidence of regenerative anaemia have been presented, with no quantitative count of reticulocytes. Moreover, no studies have correlated an increase in indirect bilirubin with the severity of haemolytic anaemia in cattle. Therefore, this study aimed to identify the causative agents of tick‐borne haemolytic anaemia in grazing cattle and determine the severity of anaplasmosis and theileriosis, which are considered benign in the ROK. In addition, we examined the changes in complete blood count (CBC), reticulocytes, indirect bilirubin and L‐lactate, which are involved in regenerative anaemia, and their correlation with anaemia severity in cattle with tick‐borne haemolytic anaemia.

## 2 MATERIAL AND METHODS

### 2.1 Animal and sampling

This study was approved by the Institutional Animal Care and Use Committee of the National Institute of Animal Science, ROK (JBNU IACUC No. NON2023‐123). All experimental procedures involving animals were conducted in strict accordance with relevant guidelines and regulations.

**FIGURE 1:**
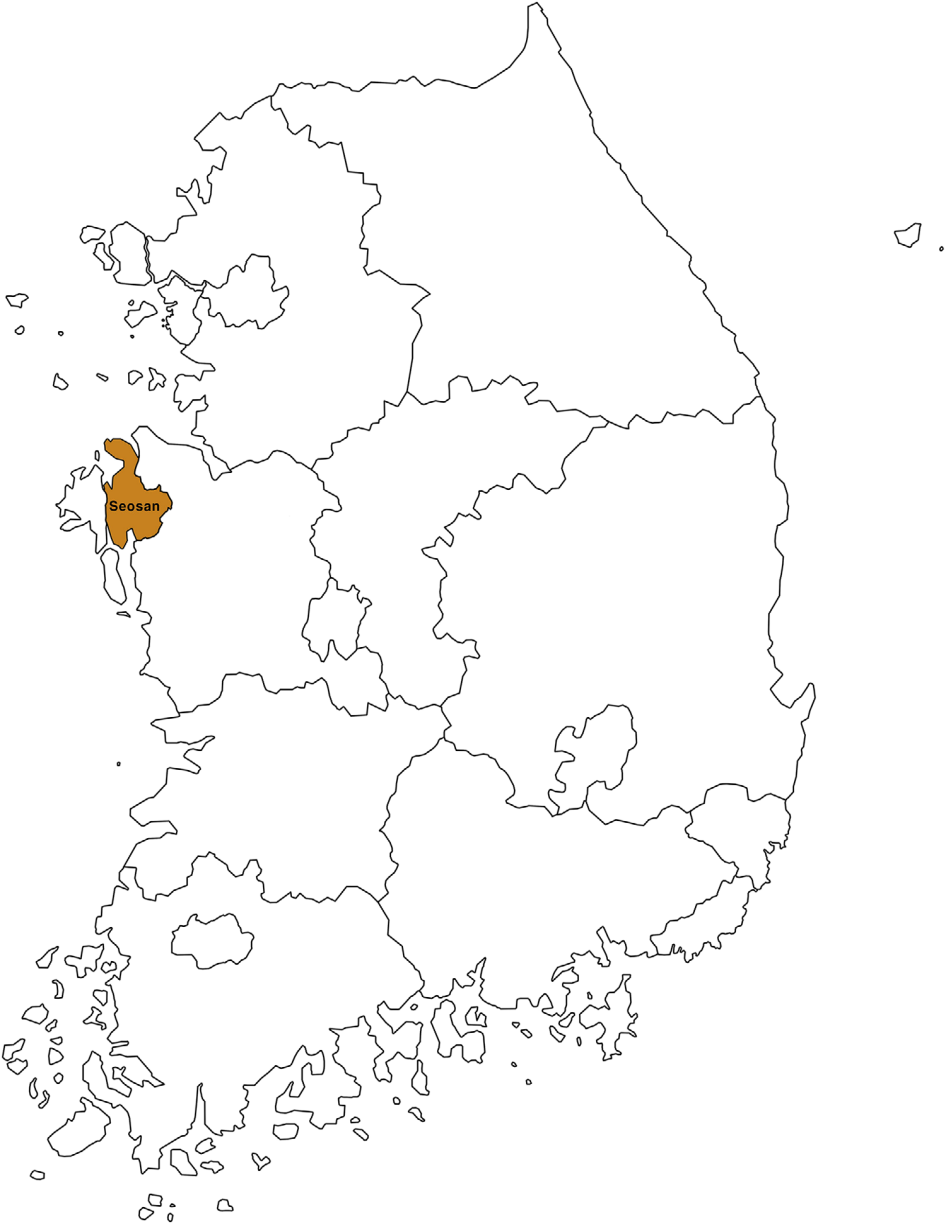
Map showing the region where blood samples were collected in the Republic of Korea.

Blood was collected from 82 Korean indigenous cattle [grazing (*n* = 67) and indoor (*n* = 15)], which were raised on one farm in the ROK (Figure 1). Pregnant Korean beef cows aged 22−48 months were selected. The grazing group was fed forages of orchard grass and tall fescue, and the indoor group was fed concentrate and roughage with a dry matter intake of 5.5 kg/day (10% crude protein). Blood samples (10 mL) were taken from the jugular vein and divided into EDTA‐supplemented tubes (BD Vacutainer^®^, Franklin Lakes, NJ, USA) and serum separation tubes (Vacutte serum tubes^®^, Greiner Bio‐One, Austria).

All samples were placed on ice and immediately transported to the laboratory. On arrival, L‐lactate was measured using a portable lactate meter (Statstrip Xpress lactate meter; Nova Biomedical, Waltham, MA, USA), and the serum was separated by centrifugation at 3000 *g* for 10 min.

Cattle in the indoor group were usually pastured in the past; however, but these animals were housed in a barn for the past year. The grazing and indoor cattle were used as the experimental and control groups, respectively.

### 2.2 CBC and serum biochemical profiles

CBC was performed using an automated haemocytometer, IDEXX ProCyte Dx (IDEXX Laboratories, Westbrook, ME, USA). Haematological analysis included the following examinations: RBC profiles, that is, RBC count, haematocrit (HCT), mean corpuscular volume (MCV), mean corpuscular haemoglobin concentration (MCHC) and reticulocytes; white blood cell (WBC) profiles, that is, total WBC count, neutrophil, lymphocyte, monocyte, eosinophil and basophil counts; and platelet counts.

LDH, total bilirubin and direct bilirubin concentrations in the serum were measured using an automated dry biochemistry analyser (FUJI DRI‐CHEM 4000i, Tokyo, Japan). Indirect bilirubin concentration was calculated as the total bilirubin concentration minus the direct bilirubin concentration. Hp concentrations were analysed using a commercial ELISA kit (PHASETM, Colorimetric Assay, Tridelta Development, Kildare, Ireland).

### 2.3 DNA extraction, PCR and sequencing analysis

Genomic DNA was extracted from each blood sample (200 µL) using the DNeasy Blood Kit (Qiagen, Valencia, California, USA) according to the manufacturer's instructions and stored at −80°C. The species‐specific primers used to amplify *T. orientalis* and *A. phagocytophilum* are listed in Table 1. PCR was performed under the following conditions: 98°C for 5 min; followed by 35 cycles of 10 s at 98°C; an annealing step at the appropriate temperature and time (Table 1), 72°C for 1 min; and a final extension at 72°C for 5 min. Distilled water was used as a negative control for all PCRs. Amplicons were separated by 1.5% agarose gel electrophoresis, visualised by staining with ethidium bromide, and photographed under UV light. All PCR products were purified using an AccuPrep^®^ PCR Purification Kit (Bioneer, Daejeon, ROK) according to the manufacturer's instructions and directly sequenced (Bioneer).

**TABLE 1:** Comparison of complete blood counts and serum biochemistry profiles between indoor and grazing cattle.

	Indoor cattle (*n* = 15)	Grazing cattle (*n* = 67)	
Parameters	mean ± SD	mean ± SD	*p* Value^*^
RBC (10^6^/µL)	7.0 ± 1.0	4.9 ± 1.3	**0.000**
HCT (%)	36.4 ± 5.3	27.1 ± 5.2	**0.000**
Hb (g/dL)	11.7 ± 1.6	8.5 ± 1.6	**0.000**
MCV (fL)	52.2 ± 6.1	57.5 ± 12.1	0.112
MCHC (g/dL)	32.3 ± 1.2	31.3 ± 1.6	**0.020**
Reticulocyte (10^3^/µL)	0.9 ± 0.6	3.2 ± 6.9	0.205
Platelet (10^3^/µL)	220 ± 54	158 ± 70	**0.002**
WBC (10^3^/µL)	15.8 ± 2.7	14.4 ± 3.1	0.102
Neutrophil (10^3^/µL)	5.6 ± 1.2	2.5 ± 1.1	**0.000**
Lymphocyte (10^3^/µL)	7.8 ± 2.2	8.4 ± 1.7	0.201
Monocyte (10^3^/µL)	0.9 ± 0.2	2.8 ± 1.0	**0.000**
Eosinophil (10^3^/µL)	1.5 ± 1.2	0.5 ± 0.4	**0.000**
Basophil (10^3^/µL)	0.1 ± 0.2	0.1 ± 0.1	0.746
Total bilirubin (mg/dL)	0.11 ± 0.0	0.33 ± 0.5	0.072
Direct bilirubin (mg/dL)	0.10 ± 0.0	0.11 ± 0.0	0.264
Indirect bilirubin (mg/dL)	0.01 ± 0.0	0.21 ± 0.4	0.065
L‐lactate (mmol/L)	0.6 ± 0.3	1.8 ± 0.7	**0.000**
Haptoglobin (mg/dL)	13.1 ± 4.4	11.9 ± 4.0	0.353

Abbreviations: HCT, haematocrit; Hb, haemoglobin; RBC, red blood cells; MCV, mean corpuscular volume; MCHC, mean corpuscular haemoglobin concentration; WBC, white blood cells.

Bold values (*P* < 0.05) were considered to be statitical significance.

* *t*‐test.

### 2.4 Statistical analysis

Statistical analyses were performed using SPSS 29.0 software package (SPSS, Chicago, Illinois, USA). The results of blood tests from indoor and grazing cattle were analysed using two‐tailed independent *t*‐test and the Mann−Whitney *U* test according to results of the normality test, and data sorted by infection status of TBPs were analysed using one‐way ANOVA and Kruskal–Wallis test. Data are presented as mean ± standard deviation, and statistical significance was set at *p* < 0.05.

## 3 RESULTS

### 3.1 Comparison of CBC and serum biochemistry results of indoor and grazing cattle

Among the RBC profiles, RBC (*p* = 0.000), HCT (*p* = 0.000), Hb (*p* = 0.000), and MCHC (*p* = 0.020) values were significantly lower in grazing cattle than in indoor cattle (Table 1). Platelet counts were also significantly lower in grazing cattle (*p* = 0.002) than in indoor cattle (Table 1). MCV and reticulocyte levels in grazing cattle greatly increased compared to than in indoor cattle; however, the differences were not statistically significant (Table 1).

WBC tests revealed marked neutropenia (*p* = 0.000), eosinopenia (*p* = 0.000), and monocytosis (*p* = 0.000) in grazing cattle (Table 1).

Although the total bilirubin values in grazing cattle increased three‐fold compared to those in indoor cattle, the difference was not statistically significant. L‐Lactate levels were significantly higher in grazing cattle than in indoor cattle (Table 1).

### 3.2 Detection of tick‐borne pathogens (TBPs)

Blood samples (*n* = 82) from the cattle were screened for TBPs using PCR; the results are presented in Table 2. The overall infection rate of TBP was 70.7% (58/82; 95% confidence interval (CI): 60.9−80.6). The prevalence of TBPs was higher in grazing cattle (82.1%, 55/67; 95% CI: 72.9−91.3) than in indoor cattle (20.0%, 3/15; 95% CI: 0.0−40.2). When comparing the infection rate of TBPs according to grazing type, co‐infection was observed in only three (20%, 3/15) indoor cattle, whereas co‐infection was detected in 29 (43.3%, 29/67) grazing cattle. In addition, single infections by *T. orientalis* or *A. phagocytophilum* were not detected in indoor cattle, whereas each single infection of *T. orientalis* and *A. phagocytophilum* was found in 25 (37.6%, 25/67) and one (1.5%, 1/67) grazing cattle, respectively. Overall, marked differences in the infection rate of TBPs were observed between indoor and grazing cattle.

**TABLE 2:** Prevalence of TBPs detected in Korean native cattle according to grazing type.

	Indoor cattle (*n* = 15)		Grazing cattle (*n* = 67)		Total (*n* = 82)	
Pathogens	No. of positive (%)	95% CI	No. of positive (%)	95% CI	No. of positive (%)	95% CI
*T. orientalis*	0 (0%)	0.0−0.0	25 (37.3%)	25.7−48.9	25 (30.5 %)	20.5−40.5
*A. phagocytophilum*	0 (0%)	0.0−0.0	1 (1.5%)	0.0−4.4	1 (1.2%)	0.0−3.6
Co‐infection	3 (20.0%)	0.0−40.2	29 (43.3%)	31.4−55.1	32 (39.0%)	28.5−49.6
Total	3 (20.0%)	0.0−40.2	55 (82.1%)	72.9−91.3	58 (70.7%)	60.9−80.6

95% CI: confidence interval.

### 3.3 Comparison of CBC and serum biochemistry results between indoor and grazing cattle according to tick‐borne infection

Haematological analyses of indoor and grazing cattle according to tick‐borne infection were compared. Grazing cattle with *A. phagocytophilum* infection showed the most significant differences in haematological parameters (Table 3). In indoor cattle, no significant differences were observed in the haematological results between the ‘no infection’ and ‘co‐infection’ groups (Table 3). In contrast, by Scheff's post‐hoc test, in grazing cattle, RBC and HCT values significantly decreased compared to those in the ‘no infection’, *T. orientalis*‐infected and co‐infection groups. Although no statistically significant differences were observed in the MCV and reticulocyte values, both increased in grazing cattle with co‐infection.

**TABLE 3:** Comparison of complete blood counts and serum biochemistry profiles between indoor and grazing cattle according to TBPs infection.

	Indoor cattle (*n* = 15)		Grazing cattle (*n* = 67)				
Parameters	No infection (*n* = 12)	Co‐infection* (*n* = 3)	No infection (*n* = 12)	*T. orientalis* (*n* = 25)	*A. phagocytophilum* (*n* = 1)	Co‐infection (*n* = 29)	*p* Value
RBC (10^6^/µL)	7.1 ± 1.0^b^	6.6 ± 0.3^b^	5.5 ± 1.1^a,b^	5.5 ± 1.0^a,b^	4.7	4.3 ± 1.4^a^	**0.000**
HCT (%)	37.0 ± 5.6^c^	33.9 ± 2.9^b,c^	30.1 ± 3.3^a,b,c^	28.9 ± 3.7^a,b^	21.0	24.6 ± 5.7^a^	**0.000**
Hb (g/dL)	12.0 ± 1.7^c^	10.7 ± 0.5^b,c^	9.2 ± 0.9^a,b^	9.1 ± 1.1^a,b^	7.1	7.7 ± 1.7^a^	**0.000**
MCV (fL)	52.4 ± 6.1	51.6 ± 5.9	56.2 ± 8.3	54.4 ± 9.9	44.7	61.1 ± 14.1	0.110
MCHC (g/dL)	32.4 ± 1.1	31.7 ± 1.2	30.7 ± 1.4	31.5 ± 1.5	0.0	31.2 ± 1.7	0.075
RET (10^3^/µL)	0.9 ± 0.7	0.9 ± 0.3	1.1 ± 1.0	2.2 ± 3.3	0.9	5.0 ± 9.7	0.130
PLT (10^3^/µL)	219 ± 37	225 ± 95	145 ± 76	191 ± 71	88	138 ± 55	**0.001**
WBC (10^3^/µL)	15.6 ±2.5	16.4 ± 3.5	13.8 ± 2.8	15.9 ± 3.0	13.8	13.3 ± 2.7	**0.010**
NEU (10^3^/µL)	5.7 ± 1.1^b^	5.0 ± 1.4^b^	2.7 ± 1.0^a^	3.0 ± 1.1^a^	3.3	2.0 ± 0.9^a^	**0.000**
LYM (10^3^/µL)	7.4 ± 1.8	9.3 ± 2.8	8.1 ± 1.4	9.3 ± 1.8	7.3	7.9 ± 1.4	**0.008**
MON (10^3^/µL)	0.9 ± 0.2	1.0 ± 0.2	2.3 ± 0.7	2.9 ± 1.0	2.5	3.0 ± 1.0	**0.000**
EOS (10^3^/µL)	1.6 ± 1.2	1.1 ± 0.5	0.6 ± 0.4	0.6 ± 0.4	0.4	0.4 ± 0.2	**0.015**
BASO (10^3^/µL)	0.1 ± 0.3	0.0 ± 0.0	0.1 ± 0.1	0.1 ± 0.1	0.4	0.1 ± 0.1	0.892
TB (mg/dL)	0.11 ± 0.0	0.10 ± 0.0	0.14 ± 0.1	0.19 ± 0.1	0.40	0.52 ± 0.6	**0.013**
DB (mg/dL)	0.10 ± 0.0	0.10 ± 0.0	0.10 ± 0.0	0.10 ± 0.1	0.10	0.12 ± 0.1	0.154
IDB (mg/dL)	0.01 ± 0.0	0.00 ± 0.0	0.04 ± 0.1	0.08 ± 0.1	0.30	0.39 ± 0.6	**0.012**
L‐lactate (mmol/L)	0.6 ± 0.3	0.8 ± 0.4	1.4 ± 0.4	1.5 ± 0.5	3.4	2.2 ± 0.7	**0.000**
Hp (mg/dL)	13.1 ± 4.9	13.1 ± 1.8	10.7 ± 3.0	10.8 ± 3.5	19.7	13.0 ± 4.2	0.190

*Note*: No infection means that TBPs were not detected.

*Cattle were co‐infected with *T. orientalis* and *A. phagocytophilum*.

Abbreviations:BASO, basophils; DB, direct bilirubin; EOS, eosinophils; HCT, haematocrit; Hb, haemoglobin; Hp, haptoglobin; IDB, indirect bilirubin; LYM, lymphocytes; MCV, mean corpuscular volume; MCHC, mean corpuscular haemoglobin concentration; MON, monocytes; NEU, neutrophils; PLT, platelets; RBC, red blood cells; RET, reticulocytes; TB, total bilirubin; WBC, white blood cells.

Bold values (*P* < 0.05) were considered to be statitical significance.

Moreover, significant neutropenia was observed in grazing cattle with co‐infection compared to that in the ‘no infection’ and *T. orientalis*‐infected groups (Table 3). Monocytes greatly increased in this group compared to that in the non‐infected group; however, the difference was not statistically significant. In addition, grazing cattle showed no significant differences in other parameters.

According to ANOVA test, there were statistically significant differences in RBC, HCT, Hb, platelet, WBC, neutrophil, eosinophil, lymphocyte, monocyte, total bilirubin, indirect bilirubin, and L‐lactate values among the five groups (Table 3)

### 3.4 Differences in TBP infections according to grazing frequency

We compared the prevalence of TBPs according to grazing frequency in grazing cattle. The overall prevalence of TBPs was 80.8% (95% CI: 70.1−91.5) in 1st‐time grazing cattle and 86.7% (95% CI: 69.5−100) in 2nd‐time grazing cattle (Table 4). Regarding infection rates, co‐infections were most frequently detected in 1st‐time grazing cattle, followed by *T. orientalis* and *A. phagocytophilum* infections. Whereas in 2nd‐time grazing cattle, *T. orientalis* infection was the most common, followed by co‐infection (Table 4). Interestingly, *A. phagocytophilum* infection markedly reduced in 2nd‐time grazing cattle compared to that in 1st‐time grazing cattle (Table 4).

**TABLE 4:** Difference in TBP occurrence according to the grazing frequency in grazing cattle.

	1st‐time grazing (*n* = 52)		2nd‐time grazing (*n* = 15)		Total (*n* = 67)	
Pathogens	No. of positive (%)	95% CI	No. of positive (%)	95% CI	No. of positive (%)	95% CI
*T. orientalis*	15 (28.8%)	16.5−41.2	10 (66.7%)	42.8−90.5	25 (37.3 %)	25.7−48.9
*A. phagocytophilum*	1 (1.9%)	0.0−5.7	0 (0%)	0.0−0.0	1 (1.5%)	0.0−4.4
Co‐infection	26 (50.0%)	36.4−63.6	3 (20.0%)	0.0−40.2	29 (43.3%)	31.4−55.1
Total	42 (80.8%)	70.1−91.5	13 (86.7%)	69.5−100	55 (82.1%)	72.9−91.3

95% CI: confidence interval.

### 3.5 Comparison of CBC and serum biochemistry results between 1st‐ and 2nd‐time grazing cattle

**FIGURE 2:**
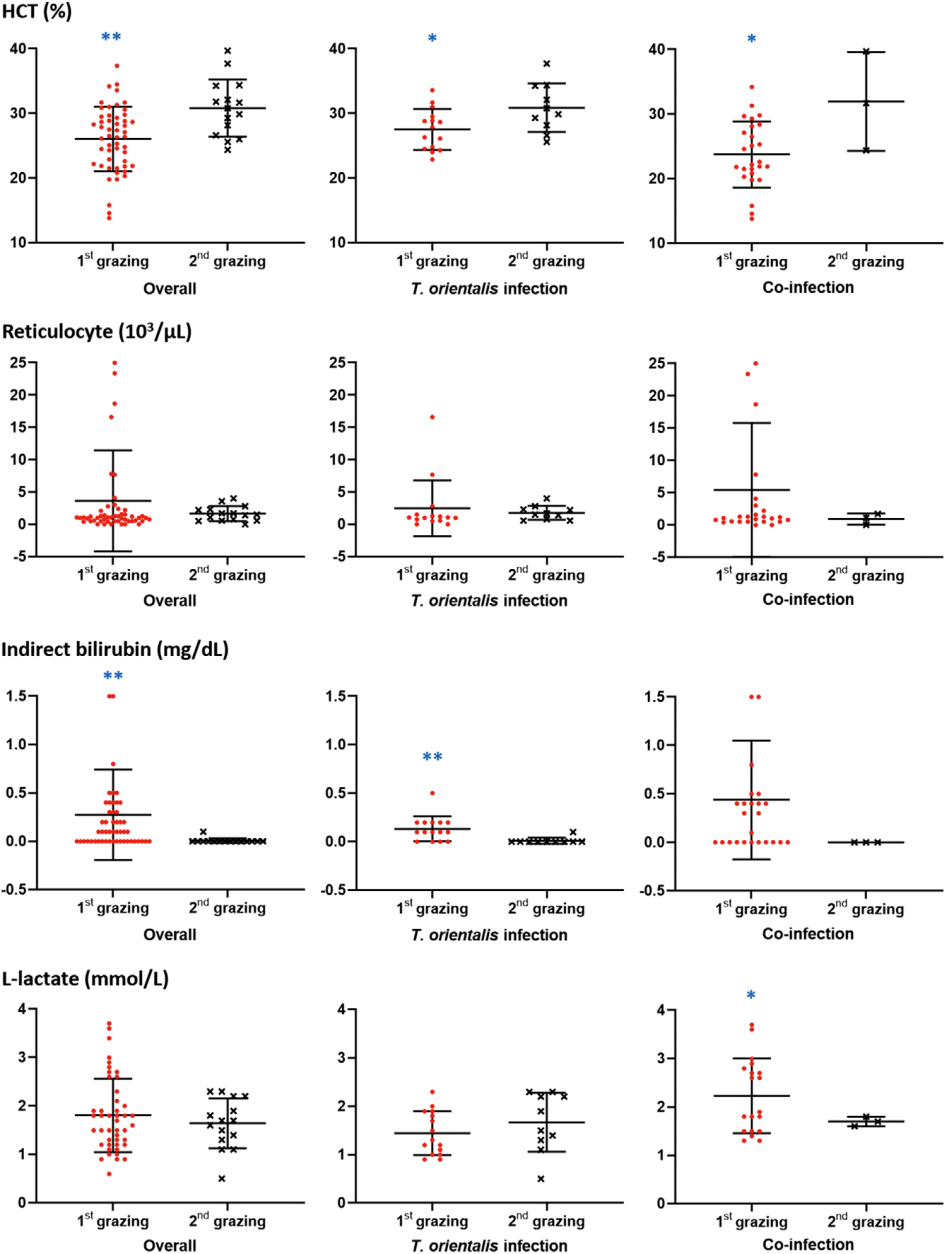
HCT, reticulocyte, indirect bilirubin and L‐lactate levels (mean ± SD) according to infection status of TBPs between 1st‐ and 2nd‐time grazing cattle. *p < 0.05, **p < 0.01.

Haematological analysis was performed in 1st‐ and 2nd‐time grazing cattle. Among these parameters, HCT, indirect bilirubin, and L‐lactate levels were significantly different between the groups. First, the HCT value significantly decreased in 1st‐time grazing cattle (26.1 ± 5.0; *p* = 0.001) compared to that in 2nd‐time grazing cattle (30.8 ± 4.4). In the case of *T. orientalis* infection, HCT significantly decreased in 1st‐time grazing cattle (27.5 ± 3.1; *p* = 0.025) compared to that in 2nd‐time grazing cattle (30.9 ± 3.6). The HCT value was lowest in 1st‐time grazing cattle with co‐infection (23.8 ± 5.0; *p* = 0.019); however, this value significantly increased in 2nd‐time grazing cattle (31.9 ± 6.2) (Figure 2).

Reticulocytes increased in 1st‐time grazing cattle than in 2nd‐time grazing cattle, and in 1st‐time grazing cattle with *T. orientalis* infection (2.5 ± 4.2; *p* = 0.628), reticulocytes also increased compared to that in 2nd‐time grazing cattle with *T. orientalis* infection (1.8 ± 1.0). Reticulocytes were highest in 1st‐time grazing cattle with co‐infection (5.4 ± 10.2); however, no statistically significant differences were observed (Figure 2).

Indirect bilirubin levels were compared based on the presence or absence of tick‐borne infection. Indirect bilirubin levels were significantly higher in 1st‐time grazing cattle (0.27 ± 0.5; *p* = 0.000) than in 2nd grazing cattle (0.01 ± 0.0). In 1st grazing cattle with *T. orientalis* infection (0.13 ± 0.1; *p* = 0.003), indirect bilirubin level also significantly increased compared with that in 2nd‐time grazing cattle with *T. orientalis* infection (0.01 ± 0.0) (Figure 2). Although indirect bilirubin levels were highest in 1st‐time grazing cattle with co‐infection (0.44 ± 0.6; *p* = 0.233) compared with that in 2nd‐time grazing cattle with co‐infection (0.00 ± 0.0), the difference was not statistically significant (Figure 2).

L‐lactate levels were lower in 1st‐time grazing cattle with *T. orientalis* infection (1.4 ± 0.4; *p* = 0.304) than in 2nd‐time grazing cattle with *T. orientalis* infection (1.7 ± 0.6). L‐lactate levels were significantly higher in 1st‐time grazing cattle with co‐infection (2.2 ± 0.8; *p* = 0.010) than in 2nd‐time grazing cattle with co‐infection (1.7 ± 0.1) (Figure 2).

### 3.6 Correlation analysis between non‐infected and co‐infected 1st‐time grazing cattle

Correlation analysis was performed for four parameters: HCT, reticulocytes, indirect bilirubin, and L‐lactate. These showed the greatest changes between non‐infected and co‐infected 1st‐time grazing cattle.

**FIGURE 3:**
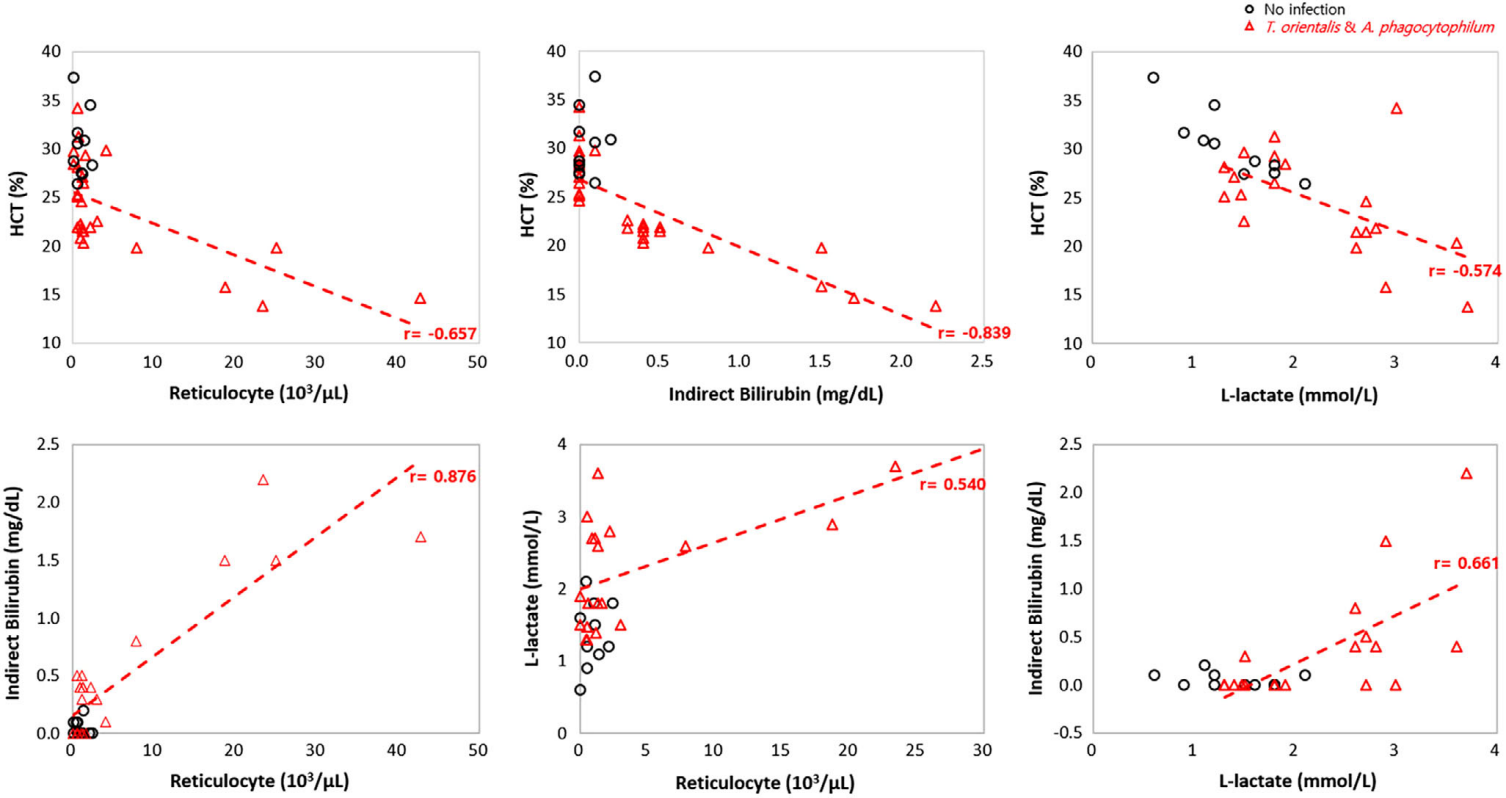
Correlation analysis of HCT, reticulocyte, indirect bilirubin, and L‐lactate between non‐infected and co‐infected in 1st‐time grazing cattle.

Compared to non‐infected cattle, a negative correlation was observed between HCT and reticulocytes (*r* = −0.657; *p* < 0.05) and between HCT and L‐lactate (*r* = −0.574; *p* < 0.05) in grazing cattle with co‐infection. Furthermore, in these cattle, a strong negative correlation (*r* = −0.839; *p* < 0.05) was found between HCT and indirect bilirubin (Figure 3).

In grazing cattle with co‐infection, a strong positive correlation (*r* = 0.876; *p* < 0.05) was observed between indirect bilirubin and reticulocytes. In addition, positive correlations between L‐lactate and reticulocytes (*r* = 0.540; *p* < 0.05) and between indirect bilirubin and L‐lactate (*r* = 0.661; *p* < 0.05) were found (Figure 3).

## 4 DISCUSSION

TBPs have been reported to cause serious health problems in ruminants, leading to substantial economic losses to the livestock industry worldwide. Among these, *T. orientalis* and *Anaplasma* spp. are the most important tick‐transmitted pathogens affecting domestic and wild ruminants in the ROK (Choi et al., 2016; Han et al., 2018, 2017; Park et al., 2018; Shin et al., 2020). Interestingly, *Anaplasma* spp. detected in this study was *A. phagocytophilum*, which was mostly co‐infected with *T. orientalis* rather than a single infection. According to our results, *T. orientalis* was mainly found in grazing cattle but not in indoor cattle. This can be explained by the limited number of indoor cattle. Furthermore, co‐infection with *T. orientalis* and *A. phagocytophilum* was more frequent in grazing cattle than in indoor cattle. Currently, the transmission route of these pathogens cannot be ascertained in indoor cattle because these cattle were housed in a barn. Our results suggest that tick exposure cannot be completely ruled out in indoor cattle.

*T. orientalis* has been reported to cause anaemia in cattle (Choi et al., 2016). However, in this study, cattle infected with *T. orientalis* alone did not have severe anaemia but mild anaemia (Choi et al., 2016; Kim et al., 2017; Park et al., 2019). This result is somewhat different from previous studies reported in other countries (Forshaw et al., 2020; Lawrence et al., 2018; McFadden et al., 2017). Based on the sequence analysis of the major piroplasm surface protein (MPSP) gene, *T. orientalis* is divided into at least 11 different genotypes globally (Sivakumar et al., 2014). Of the 11 genotypes, type 2 (Ikeda) is known to be associated with severe disease (Kamau et al., 2011). Since sequencing analysis was not performed for MPSP‐positive samples, the exact genotypes were not determined. This may explain the different results between studies. Anaemia was more severe in grazing cattle co‐infected with *T. orientalis* and *A. phagocytophilum* than those infected with *T. orientalis* alone. Although we cannot draw an exact conclusion because only one animal was infected with *A. phagocytophilum*, this animal exhibited severe anaemia. These results indicated that *A. phagocytophilum* infection is strongly associated with anaemia. To the best of our knowledge, this is the first study to report anaemia caused by *A. phagocytophilum* infection in cattle. Although the incidence of *A. phagocytophilum* infection is increasing in the ROK, its pathogenicity remains unclear. Thus, further studies are required to determine the association between anaemia and *A. phagocytophilum*.

Anaemia is generally classified based on MCV and MCHC values (Jain et al., 1990; Jones & Allison, 2007; Schalm et al., 2010; Stockham & Scott, 2008b). In grazing cattle with co‐infection, the MCV value increased; however, MCHC values were not significantly different from that in the ‘no infection’ group. In this study, most cattle with anaemia had macrocytic normochromic anaemia, whereas grazing cattle with co‐infection had macrocytic hypochromic anaemia. The more severe the haemolytic anaemia, the higher the number of immature RBCs. This is consistent with the increase in reticulocytes observed in this study, although there was no significant difference between the ‘no infection’ and co‐infection groups. In cattle, regenerative anaemia can be confirmed by the presence of polychromasia or reticulocytes on a blood smear as well as basophilic stippling or Howell‐Jolly bodies (Jain et al., 1990; Jones & Allison, 2007; Schalm et al., 2010; Stockham & Scott, 2008b). In particular, reticulocytosis is considered a sensitive indicator of regenerative anaemia in cattle. Several studies have reported that during regenerative anaemia, reticulocytes are known to increase six‐ to eight‐fold in dogs and three‐ to five‐fold in cats; however, the increase in reticulocytes is not high in cattle (Stockham & Scott, 2008b). Nevertheless, in this study, the number of reticulocytes greatly increased in cattle with co‐infection, compared to those with only *T. orientalis* infection. Our findings suggest that in grazing cattle with co‐infection, the relative increase in reticulocytes may be caused by a decrease in normal RBCs. Therefore, the corrected reticulocyte percentage (CRP) is required to determine whether the increase in reticulocytes is due to regenerative anaemia (Schalm et al., 2010; Stockham & Scott, 2008b). The mean CRP (11.8 ± 3.37 × 10^3^ cells/µL) in the six grazing cattle with co‐infection showing significantly increased reticulocyte count was very high compared to that of the indoor group (0.9 ± 0.3 × 10^3^ cells/µL). Consequently, it can be assumed that the calculated CRP levels increased due to regenerative anaemia. Therefore, reticulocyte production index in cattle should be investigated in future studies.

Once haemolysis occurs, RBCs are lysed, and Hb and LDH are released into the blood, resulting in elevated serum LDH and haemoglobinemia (Schalm et al., 2010; Stockham & Scott, 2008a; Stockham & Scott, 2008b; Zachary & McGavin, 2017). Owing to the cytotoxic nature of free Hb, it binds to Hp and travels to the spleen, where it is phagocytosed by macrophages (Schaer et al., 2014). Thus, a decrease in Hp is observed. However, in this study, Hp slightly increased in grazing cattle with co‐infection compared to that in cattle with no infection, and the concentration was similar to that of indoor cattle. As a result, the amount of Hp in these cattle was not sufficient to bind to Hb, and the remaining (free) Hb was transported to the kidneys, where it was broken down. In degraded Hb, haeme is converted to bilirubin, which is bound to albumin and transported to the liver (Schalm et al., 2010; Stockham & Scott, 2008a; Stockham & Scott, 2008b; Zachary & McGavin, 2017). This leads to an increase in indirect bilirubin levels, which is consistent with the current results.

In this study, mild neutropenia and monocytosis were observed in grazing cattle with co‐infection. *A. phagocytophilum* infects granulocytes, particularly neutrophils (Atif, 2015), and the infection is generally characterised by leukopenia and thrombocytopenia. Grazing cattle with co‐infection exhibited significant neutropenia and leukopenia compared to those infected with only *T. orientalis*. In particular, the band form of neutrophils was observed in cattle with anaemia, supporting the idea that neutrophil destruction occurs without reducing the pool of stored neutrophils. As neutrophils and monocytes share the same precursor cells, monocytes tend to increase as neutrophils decrease (Schalm et al., 2010; Stockham & Scott, 2008b). Moreover, thrombocytopenia occurred more frequently in grazing cattle with co‐infection than in grazing cattle with only *T. orientalis* infection. This can be explained by the presence of *A. phagocytophilum*.

We found that the prevalence of TBPs in 1st‐ and 2nd‐time grazing cattle differed according to grazing frequency. Interestingly, the infection rate of *A. phagocytophilum* markedly reduced in 2nd‐time grazing cattle compared to that in 1st‐time grazing cattle. These results show that grazing frequency affects *A. phagocytophilum* infection. When put out to pasture for the first time, cattle appear to be highly susceptible to *A. phagocytophilum*. This may be due to the antibodies produced after *A. phagocytophilum* infection during 1st‐time grazing, which then act during 2nd‐time grazing. In contrast, a previous study showed that antibodies against *T. orientalis* can persist for 3−5 years without reinfection in cattle that have recovered from infection (Delves & Roitt, 1998). Moreover, haemolytic anaemia was more severe in the 1st‐time grazing group than in the 2nd‐time grazing group. Contrary to our expectations, our findings demonstrated that severe anaemia was caused by co‐infection with *T. orientalis* and *A. phagocytophilum* compared to that of single infection with *T. orientalis*, indicating that *A. phagocytophilum* infection is significantly associated with severe anaemia. These results highlight the importance of *A. phagocytophilum* in grazing cattle.

The diagnosis of anaemia in cattle is based on the HCT value, which is classified as mild (20−26%), moderate (14−19%), severe (10−13%) and very severe (<10%) (Jones & Allison, 2007; Metivier et al., 2000). More severe haemolytic anaemia may require blood transfusion, and the primary measure used in clinical practice is the HCT, with an HCT of 14% or less recognised as a panic value (Hardy, 2004; Metivier et al., 2000). It has also been reported that a venous blood oxygen tension of 25 mmHg or less, a lactate concentration of 4 mmol/L or more, and the degree of metabolic acidosis can be used as cofactors for transfusion requirements in large animals (Hardy, 2004). Because anaemia‐induced metabolic acidosis is caused by accumulation of lactate in response to tissue hypoxia, we measured whole blood L‐lactate rather than venous blood pH or anion gap in this study. However, in our study, L‐lactate levels were higher in the anaemic population than in the control group. More importantly, we found a negative correlation between L‐lactate and HCT levels (*r* = 0.574, *p* < 0.05). L‐lactate test may be an adequate adjunct test to assess anaemia severity. Further research is needed to identify L‐lactate as a panic value predictor of cattle that require blood transfusion.

## AUTHOR CONTRIBUTIONS

YJ Kim, KS Choi, and JH Park conceived and designed this study. YJ Kim, JY Ku, YW Jung, YH Lim, MJ Ji, YJ Park, and HC Cho and performed the experiments and, and helped interpret the results. YJ Kim, KS Choi, and JH Park wrote the paper. All authors have read and agreed to the published version of the manuscript.

## CONFLICT OF INTEREST STATEMENT

The authors declare that they have no competing interest.

### PEER REVIEW

The peer review history for this article is available at https://publons.com/publon/10.1002/vms3.1434.

## ETHICS STATEMENT

This research was approved by the Institutional Animal Care and Use Committee (IACUC) at the National Institute of Animal Science, the Republic of Korea (JBNU IACUC No. NON2023‐123). All experimental procedures involving animals were conducted in strict accordance with relevant guidelines and regulations.

## Data Availability

All data generated or analysed during this study are included in the article. Raw data are available upon reasonable request to the corresponding author.
